# Proximal pulmonary artery wall shear stress derived from computational fluid dynamics: A noninvasive biomarker for CTEPH and perfusion mismatch

**DOI:** 10.14814/phy2.70664

**Published:** 2025-11-16

**Authors:** Yahia Bellouche, Cécile Tromeur, Florent Le Ven, Philippe Robin, Christophe Gut‐Gobert, Pierre‐Yves Le Roux, Michel Nonent, Alexandre Fauché, Jamal Elhasnaoui, Aurélia Le Hir, Marie Guegan, Christophe Leroyer, Pierre‐Yves Salaün, Bastien Pasdeloup, Romain Didier, Francis Couturaud

**Affiliations:** ^1^ Département de Cardiologie Centre Hospitalier Universitaire Brest Brest France; ^2^ Univ Brest, INSERM U1304‐GETBO, CIC INSERM 1412 Brest France; ^3^ Département de Pneumologie Centre Hospitalier Universitaire Brest Brest France; ^4^ FCRIN INNOVTE Network Saint‐Etienne France; ^5^ Département de médecine nucléaire Centre Hospitalier Universitaire Brest Brest France; ^6^ Département de Radiologie Centre Hospitalier Universitaire Brest Brest France; ^7^ Centre hospitalier de Brest Unité de Recherche Clinique Brest France; ^8^ UMR CNRS Lab‐STICC, Télécom Bretagne Plouzané France

**Keywords:** chronic thromboembolic pulmonary hypertension, computational fluid dynamics, perfusion mismatch, pulmonary artery pressure, time‐averaged wall shear stress

## Abstract

Chronic thromboembolic pulmonary hypertension (CTEPH) is a severe complication of pulmonary embolism (PE), often diagnosed late due to nonspecific symptoms and limitations of current screening tools like V/Q scintigraphy. This study investigated whether computational fluid dynamics (CFD)‐derived hemodynamic parameters, specifically time‐averaged wall shear stress (TAWSS) and oscillatory shear index (OSI), in the proximal pulmonary arteries could serve as noninvasive biomarkers for CTEPH and chronic thromboembolic disease without pulmonary hypertension (CTEPD, non‐CTEPH with V/Q mismatches). We retrospectively analyzed 90 patients (30 CTEPH, 30 CTEPD, and 30 controls without mismatch) using patient‐specific 3D CFD models reconstructed from CTPA, with RCR boundary conditions tuned to RHC data. We found significantly reduced median TAWSS in CTEPH (16.5 dyn/cm^2^) and CTEPD (27.5 dyn/cm^2^) groups compared to controls (42.0 dyn/cm^2^) (*p* < 0.001), with TAWSS also significantly lower in CTEPH versus CTEPD. OSI showed no significant inter‐group differences. Importantly, TAWSS exhibited a strong inverse correlation with V/Q mismatch status (*ρ* = −0.673, *p* < 0.001). ROC analysis revealed that TAWSS accurately predicted perfusion mismatches (AUC = 0.918), with an optimal cutoff of 27.0 dyn/cm^2^ yielding 100.0% specificity and 70.0% sensitivity. These findings demonstrate that CFD‐derived proximal pulmonary artery TAWSS is a promising noninvasive indicator of chronic thromboembolic burden, including subclinical perfusion abnormalities, offering a potential tool to enhance early detection and management of CTEPH.

## 1 INTRODUCTION

Pulmonary embolism (PE) is a major cause of global cardiovascular morbidity and mortality. In the United States, PE is estimated to cause up to 300,000 deaths annually, with sudden death frequently representing the initial clinical manifestation (Wendelboe & Raskob, 2016). In Europe, the estimated annual mortality attributable to venous thromboembolism (VTE), which includes both PE and deep vein thrombosis (DVT), exceeds 370,000 cases. Notably, 34% of deaths occur within hours of symptom onset, often before initiation of treatment, and PE remains undiagnosed until postmortem in the majority of such cases (Cohen et al., 2007). Epidemiological data indicate an annual incidence of PE ranging from 39 to 115 per 100,000 population (Wendelboe & Raskob, 2016). Although contemporary management strategies and guideline‐adherent therapies have improved outcomes, the potential for overdiagnosis—particularly of subsegmental PE—may artifactually reduce case fatality rates. The economic burden is substantial, with annual direct and indirect healthcare costs related to VTE in the European Union estimated at €8.5 billion (Barco et al., 2016). These findings underscore the clinical and public health importance of VTE and the anticipated increase in its global burden.

Chronic thromboembolic pulmonary hypertension (CTEPH), a severe complication of pulmonary embolism (PE), is characterized by non‐resolving pulmonary artery obstruction by organized thrombi, leading to sustained pulmonary hypertension. Reported post‐PE CTEPH incidence varies widely from 0.1% to 9.1%, reflecting heterogeneous populations and diagnostic strategies (Konstantinides et al., 2020). Contemporary registry‐based estimates suggest an annual CTEPH incidence of 3.1–6.0 and a prevalence of 25.8–38.4 cases per million adults (Leber et al., 2021). The number of diagnosed cases is increasing, likely due to improved recognition and expanded screening in dyspnoeic post‐PE patients and those with established risk factors. Pulmonary endarterectomy (PEA) remains the definitive treatment for operable disease, offering curative potential (Hsieh et al., 2018; Humbert et al., 2022). Nonetheless, diagnosis is often delayed due to nonspecific early symptoms. Current European Society of Cardiology (ESC) and European Respiratory Society (ERS) guidelines recommend structured follow‐up following PE, incorporating clinical evaluation, imaging, and functional assessment to facilitate early detection. Ventilation/perfusion (V/Q) scintigraphy remains the recommended initial imaging modality, given its high sensitivity and specificity for identifying CTEPH‐specific perfusion abnormalities. However, its widespread application is limited by cost, availability, and need for specialized infrastructure, particularly in non‐tertiary settings. Accordingly, more accessible and scalable diagnostic strategies are required to support timely diagnosis and intervention in CTEPH.

Advancements in computational fluid dynamics (CFD) offer novel approaches for noninvasive assessment of cardiovascular hemodynamics. CFD facilitates detailed simulation of blood flow patterns within the vascular system, providing access to parameters such as wall shear stress (WSS) and oscillatory shear index (OSI), both implicated in vascular remodeling and disease progression (Bellouche et al., 2025; Lee, 2011; Morris et al., 2016). Within the context of pulmonary hypertension, integrating CFD with clinical data from echocardiography, right heart catheterization (RHC), and computed tomography pulmonary angiography (CTPA) can elucidate hemodynamic alterations associated with chronic thromboembolic pulmonary hypertension (CTEPH). These simulations hold potential for identifying biomechanical markers indicative of CTEPH presence and severity. Time‐averaged wall shear stress (TAWSS) and OSI in the proximal pulmonary arteries have emerged as candidate biomarkers for CTEPH (Kachabi et al., 2024). Alterations in these parameters may reflect pathological changes in blood flow dynamics, offering a noninvasive method to evaluate disease risk and progression. Studies have shown reduced TAWSS and elevated OSI in CTEPH patients, suggesting disturbed flow patterns could contribute to disease pathogenesis (Bordones et al., 2018; Kachabi et al., 2024; Shi et al., 2025).

This study aims to quantify TAWSS and OSI in the proximal pulmonary arteries across three distinct post‐pulmonary embolism (PE) cohorts: patients diagnosed with CTEPH, patients without CTEPH but exhibiting perfusion mismatches on ventilation/perfusion (V/Q) scintigraphy (chronic thromboembolic disease without pulmonary hypertension [CTEPD]), and patients without CTEPH or perfusion mismatches. Additionally, this investigation seeks to determine the predictive utility of proximal pulmonary artery TAWSS in identifying perfusion mismatches, thereby assessing its potential as a noninvasive CTEPH screening tool.

## 2 MATERIALS AND METHODS

### 2.1 Study design and data collection

This retrospective case–control study, conducted at Brest University Hospital, included three groups of 30 patients each, all with a documented history of pulmonary embolism (PE) within the preceding 3–6 months. Group 1 comprised patients diagnosed with chronic thromboembolic pulmonary hypertension (CTEPH) based on 2022 European Society of Cardiology/European Respiratory Society (ESC/ERS) guidelines (Humbert et al., 2022). These patients exhibited a mean pulmonary artery pressure (mPAP) >20 mmHg, pulmonary vascular resistance (PVR) >2 Wood units, and a pulmonary artery wedge pressure (PAWP) <15 mmHg, all confirmed via right heart catheterization (RHC), alongside imaging evidence of chronic thromboembolic disease. Group 2 included patients with CTEPD, that is, patients without CTEPH but demonstrating perfusion mismatches on ventilation/perfusion (V/Q) scintigraphy, defined as areas of reduced or absent perfusion with preserved ventilation. Group 3 consisted of patients without CTEPH or V/Q scintigraphy perfusion mismatches.

Inclusion criteria for all cohorts were: age >18 years, a documented history of acute PE, completion of at least 3 months of effective anticoagulation therapy, and persistent dyspnoea despite treatment. Exclusion criteria encompassed: incomplete imaging or hemodynamic data, and significant comorbidities affecting pulmonary circulation (e.g., severe chronic obstructive pulmonary disease, interstitial lung disease). Retrospective data collection from medical records included patient demographics, clinical history, RHC parameters (mPAP, PVR, cardiac output), transthoracic echocardiography findings, computed tomography pulmonary angiography (CTPA) results, and V/Q scintigraphy images.

**FIGURE 1:**
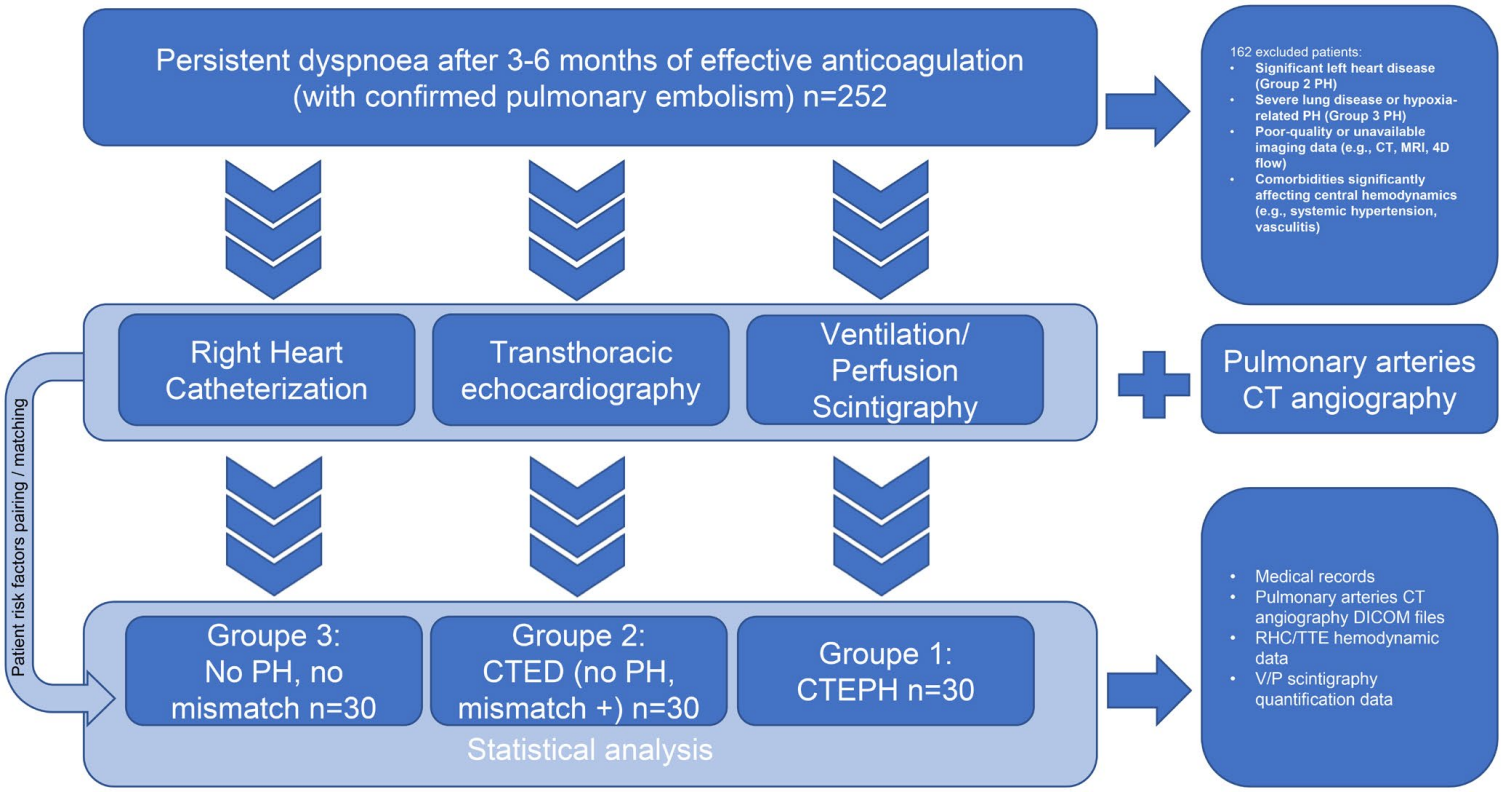
Study design and patient cohorts. Patients with persistent dyspnoea post‐PE were evaluated using RHC, TTE, V/Q scintigraphy, and CT angiography, then classified into CTEPH, CTED, or control groups (*n* = 30 each) for analysis. CT, computed tomography; PE, pulmonary embolism; RHC, right heart catheterization; TTE, transthoracic echocardiography; V/Q, ventilation/perfusion.

The study protocol (Figure 1) received approval from the local Ethics Committee of Brest University Hospital. Given the retrospective and anonymized nature of the study, the requirement for individual informed consent was waived in accordance with the French MR004 reference methodology. All procedures adhered to the ethical standards outlined in the Declaration of Helsinki.

### 2.2 Computational simulation setting

#### 2.2.1 Three‐dimensional pulmonary artery model generation

For each of the 90 patients, three‐dimensional (3D) geometric models of the proximal pulmonary artery tree, including the main pulmonary artery (MPA), left pulmonary artery (LPA), right pulmonary artery (RPA), and their major branches, were reconstructed. This process used Digital Imaging and Communications in Medicine (DICOM) files derived from computed tomography pulmonary angiography (CTPA) scans, which were acquired using standard clinical protocols with intravenous contrast to ensure optimal vascular visualization. All four exams (TTE, RHC, scintigraphy, and CTPA) were performed after 3–6 months of anticoagulation.

**FIGURE 2:**
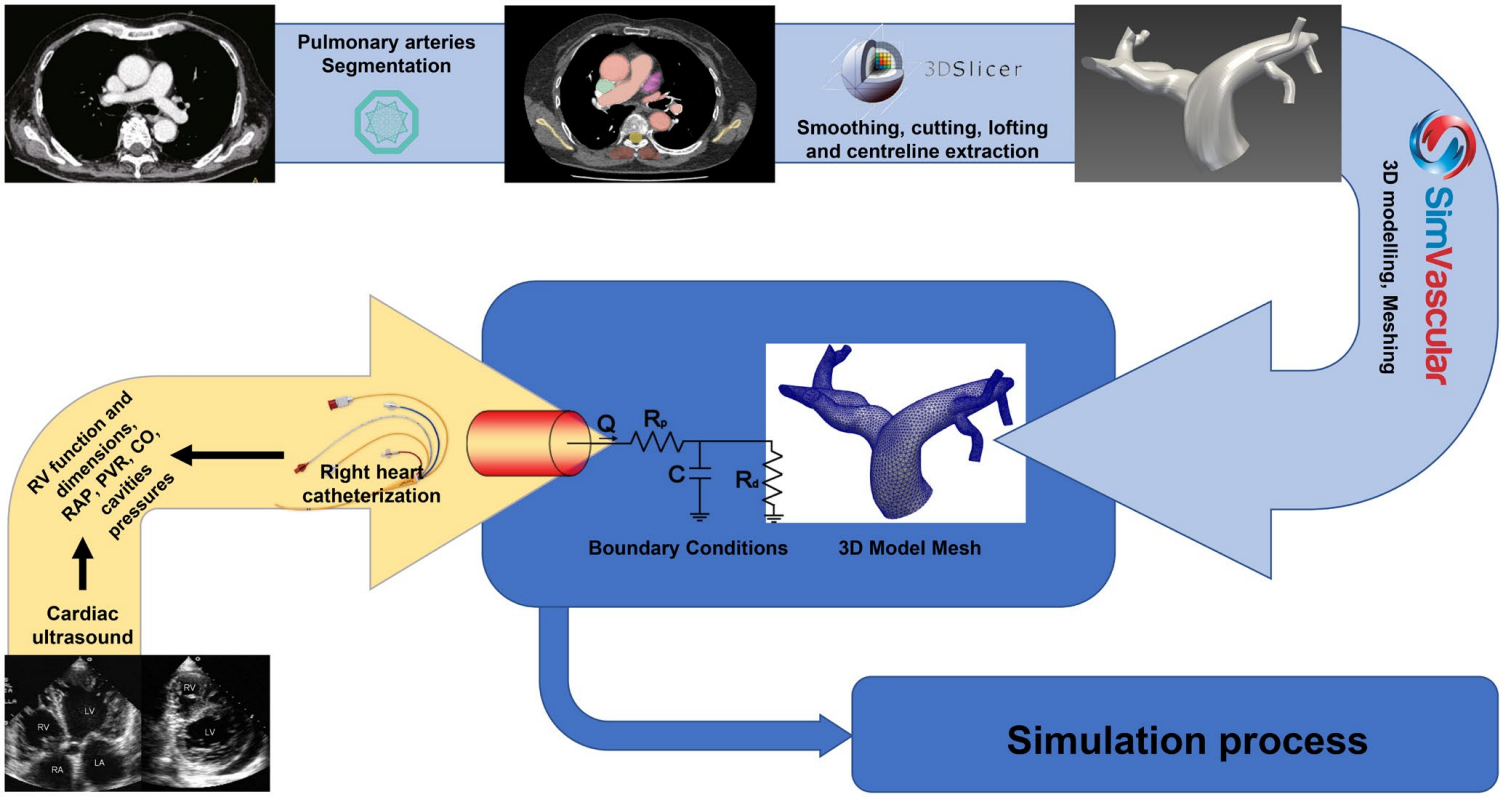
Three‐dimensional pulmonary artery model reconstruction pipeline. Pulmonary artery segmentation, 3D models reconstruction (3D Slicer), and meshing (SimVascular) were combined with patient‐specific boundary conditions derived from cardiac ultrasound and right heart catheterization. CFD, computational fluid dynamics; CO, cardiac output; PAP, pulmonary artery pressure; PCWP, pulmonary capillary wedge pressure; RAP, right atrial pressure; RV, right ventricle.

Segmentation of the pulmonary arteries from the CTPA images was performed using the MONAI deep learning setup integrated within the open‐source medical image analysis software Slicer 3D. A pretrained deep learning model from MONAI was used within the Slicer 3D environment for vessel segmentation (Diaz‐Pinto et al., 2024; Fedorov et al., 2012). The proximal pulmonary arteries were manually cropped from the CT volume to reduce computational load and focus on relevant anatomy. The model then automatically segmented the vessels, followed by manual refinement in Slicer 3D to ensure anatomical accuracy. Smoothing algorithms were applied to the segmented surfaces to generate anatomically and computationally stable 3D models of the proximal pulmonary artery tree. Following 3D model generation, vessel centreline extraction was performed on each model using a specialized module within Slicer 3D. This process generated a central path for each vessel segment, critical for defining flow directions and for subsequent hemodynamic parameter analysis (Figure 2).

#### 2.2.2 Mesh generation

The reconstructed 3D surface models were subsequently imported into the SIMVASCULAR software pipeline, an open‐source platform designed for cardiovascular modeling and simulation (Updegrove et al., 2017). Within SIMVASCULAR, a volumetric mesh, composed of interconnected tetrahedral elements, was generated for each patient‐specific pulmonary artery model using the integrated TetGen meshing library. Tetrahedral meshes are particularly suitable for complex geometries, such as the branching pulmonary arteries, facilitating accurate representation of the vascular lumen. We generated the computational mesh by setting a global maximum edge length, applying finer resolution at complex regions like bifurcations to better capture local flow dynamics along with boundary layer meshing. Mesh quality was evaluated using criteria such as minimum solid angle and aspect ratio to ensure suitability for CFD simulations. Importantly, a mesh independence test was performed to confirm the robustness of our results, as detailed in the Appendix.

#### 2.2.3 Application of boundary conditions

Computational fluid dynamics (CFD) simulations require robust boundary conditions to accurately represent the physiological environment. In this study, a patient‐specific pulsatile flow waveform, derived from the cardiac output and heart rate obtained during right heart catheterization (RHC) for each patient, was imposed at the main pulmonary artery (MPA) inlet. At the outlets of the reconstructed pulmonary artery tree, a three‐element Windkessel (RCR) model was applied to represent the lumped impedance of the distal pulmonary vasculature. The RCR model consists of a proximal resistance (R_p_), compliance (C), and a distal resistance (R_d_).

**FIGURE 3:**
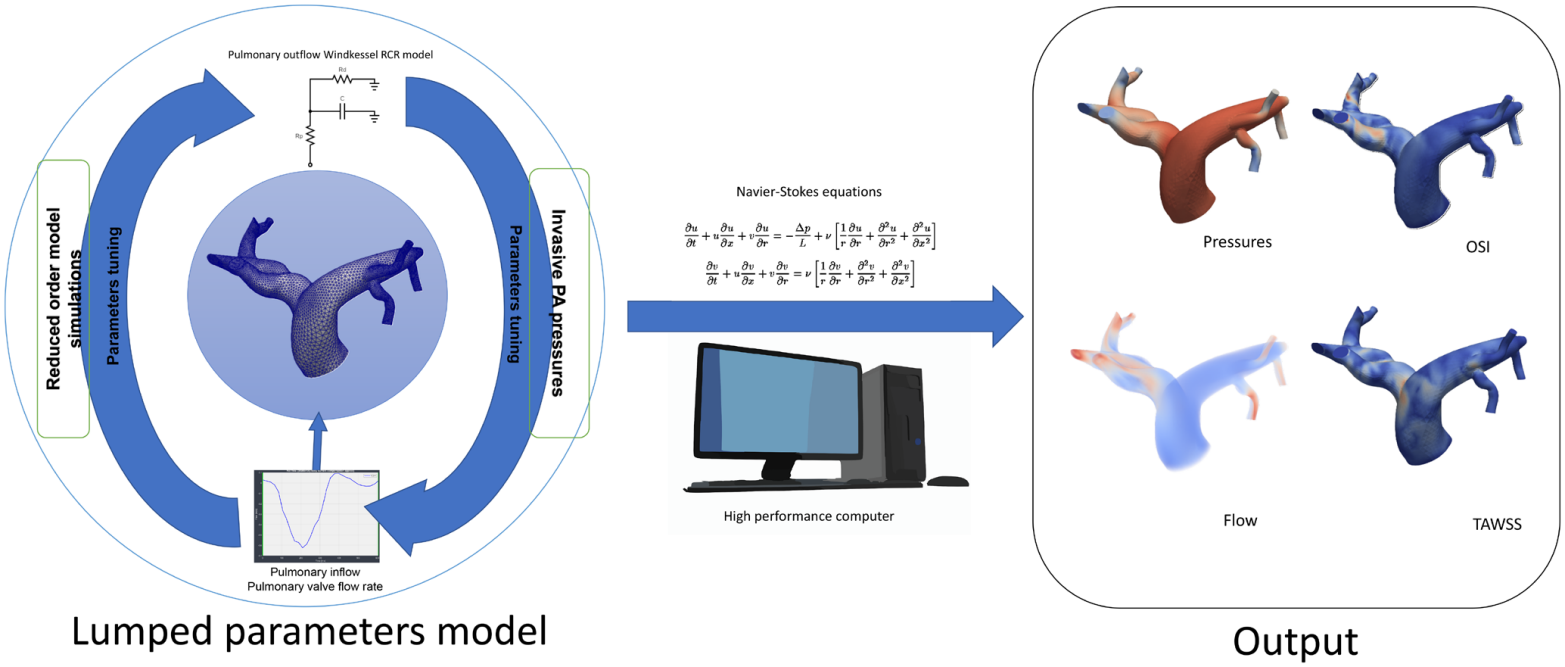
Computational fluid dynamics setup and boundary condition tuning. Patient‐specific inflow and invasive pulmonary artery pressures are used to tune a Windkessel boundary condition model. Navier–Stokes equations are solved using high‐performance computing to obtain pressure, flow, time‐averaged wall shear stress (TAWSS), and oscillatory shear index (OSI) distributions. OSI, oscillatory shear index; PA, pulmonary artery; TAWSS, time‐averaged wall shear stress.

The parameters for these RCR models were tuned through a reduced‐order simulation approach to achieve physiologically accurate pressure and flow dynamics matching the clinical RHC/TTE data. Specifically, the total pulmonary vascular resistance (PVR) was distributed among the individual outlets of the 3D model. This resistance distribution was based on a Murray's Law coefficient of 2.25 to account for microcirculatory elements, where the resistance of each outlet was inversely proportional to its diameter raised to the power of 2.25 (i.e., R outlet ∝1/D 2.25). Similarly, the total compliance of the distal pulmonary circulation was distributed among the outlets proportionally to the square of their respective diameters (i.e., C outlet∝D2), reflecting the capacitive properties of the distal vasculature. The R_p_, C, and R_d_ values at each outlet were then iteratively adjusted using a 0D‐1D simulation framework until the simulated mean pulmonary artery pressure (mPAP), PVR, and cardiac output (CO) closely corresponded to the patient‐specific measurements obtained during RHC. This diameter‐based distribution and subsequent tuning process ensure that the CFD models accurately reflect the global hemodynamic load imposed by the distal pulmonary circulation for each individual patient cohort, enhancing the physiological relevance of the calculated flow parameters within the 3D model (Figure 3).

#### 2.2.4 Computational fluid dynamics simulations and post‐processing

The governing equations for blood flow, specifically the steady‐state Navier–Stokes equations, were solved using the finite element method within the SIMVASCULAR flow solver (svSolver). Blood was modeled as an incompressible, Newtonian fluid with a constant viscosity of 0.0035 Pa·s and a density of 1060 kg/m^3^. Simulations were performed for six cardiac cycles to ensure numerical stability and capture the pulsatile nature of blood flow; data were extracted from multiple time points within the final cycle. Convergence criteria were rigorously applied to guarantee the accuracy and stability of the numerical solutions.

Post‐processing of the simulation results was conducted using the open‐source visualization software ParaView (Kitware, Clifton Park, NY, USA). This involved quantifying key hemodynamic parameters on the endothelial surface of the pulmonary arteries, including pressure distributions, blood flow velocities, and wall shear stress (WSS). Time‐averaged wall shear stress (TAWSS) was calculated by averaging the magnitude of the WSS vector over the final cardiac cycle. The oscillatory shear index (OSI) was also computed to quantify the directional changes in WSS throughout the cardiac cycle. Detailed equations for TAWSS and OSI are provided in the Appendix.

### 2.3 Statistical analysis

All statistical analyses were conducted using R Statistical Software (v4.4.1; https://ropensci.org/blog/2021/11/16/how‐to‐cite‐r‐and‐r‐packages/), with a two‐tailed *p*‐value of less than 0.05 considered statistically significant. Continuous variables were assessed for normality using the Shapiro–Wilk test. Variables exhibiting a normal distribution were summarized as mean ± standard deviation (SD), while non‐normally distributed variables were presented as median with interquartile range (IQR). Categorical variables were expressed as absolute counts and corresponding percentages.

To evaluate differences in time‐averaged wall shear stress (TAWSS) and oscillatory shear index (OSI) across the three patient groups, the Kruskal–Wallis H test was employed, given the non‐normal distribution of these variables. Upon identifying significant differences, post hoc pairwise comparisons were conducted using Dunn's test with Bonferroni correction to adjust for multiple comparisons. The relationship between TAWSS and the degree of V/Q mismatch was examined using Spearman's rank correlation coefficient, appropriate for assessing associations between non‐normally distributed continuous variables. To determine the predictive capacity of TAWSS for identifying V/Q mismatch, receiver operating characteristic (ROC) curve analysis was performed. The area under the ROC curve (AUC) was calculated to quantify the overall discriminative ability of TAWSS. The optimal cutoff value for TAWSS was identified using Youden's J statistic, which maximizes the sum of sensitivity and specificity. At this cutoff, sensitivity, specificity, positive predictive value (PPV), and negative predictive value (NPV) were computed to evaluate the diagnostic performance of TAWSS in predicting V/Q mismatch. Confidence intervals for these metrics were calculated to assess their precision.

## 3 RESULTS

### 3.1 Clinical and demographic characteristics

A total of 90 patients were included in the analysis (30 patients in each group). The median age was 67.5 years (IQR 62.0–78.5) in the CTEPH group, 67.5 (56.3–77.8) in the mismatch group, and 71.0 (63.5–76.0) in the no mismatch group (*p* = 0.788). The proportion of NYHA functional class II was 75.0% in the CTEPH group, 73.3% in the mismatch group, and 78.3% in the no mismatch group (*p* = 0.918). Associated deep vein thrombosis (DVT) was present in 65.0% of CTEPH patients, 56.7% of mismatch patients, and 47.8% of no mismatch patients (*p* = 0.526). History of pulmonary disease was recorded in 35.0%, 30.0%, and 17.4% of patients in the three respective groups (*p* = 0.398). Genetic thrombophilia was observed in 2%, 10.0%, and 4.3%, respectively (*p* = 0.301). The 6‐min walk distance (6MWD) was available for CTEPH and mismatch groups, with median values of 390.0 m (IQR 300.0–492.5) and 422.5 m (322.3–557.5), respectively (Table 1).

**TABLE 1:** Clinical characteristics of the study population.

Characteristic	CTEPH (*n* = 30)	No CTEPH with mismatch (*n* = 30)	No CTEPH nor mismatch (*n* = 30)	*p* Value
Age, years (median, IQR)	67.5 (62.0–78.5)	67.3 (56.3–77.8)	68.0 (63.5–76.0)	0.788
Sex, male *n* (%)	14 (46%)	13 (43%)	16 (53%)	0.833
BMI, kg/m^2^ (median, IQR)	26.62 (24.18–29.79)	27.55 (28.12–30.65)	26.57 (23.76–29.91)	0.26
NYHA Class II, %	75.0	73.3	78.3	0.918
NYHA Class III, %	25.0	26.7	21.7	0.918
Associated DVT, %	65.0	56.7	47.8	0.526
History of pulmonary disease, %	35.0	30.0	17.4	0.398
Genetic disorder, %	0.0	3.0	2.3	0.681
Risk factors, %	65.0	43.3	0	N/A
Mismatch V/Q (median, IQR)	0.40 (0.30–0.40)	0.30 (0.21–0.35)	N/A	0.25
NT‐proBNP, ng/L (median, IQR)	805.0 (114.3–1202.5)	657.5 (375.0–1146.3)	210.0 (118.0–257.5)	<0.001
6MWD, m (median, IQR)	390.0 (300.0–492.5)	422.5 (322.3–557.5)	N/A	N/A

Abbreviations: 6MWD, 6‐min walk distance; BMI, body mass index; CTEPH, chronic thromboembolic pulmonary hypertension; DVT, deep vein thrombosis; NT‐proBNP, N‐terminal pro–B‐type natriuretic peptide; NYHA, New York Heart Association; V/Q: ventilation‐perfusion.

NT‐ProBNP levels differed between groups: 805 ng/L (IQR 114.3–1202.5) in CTEPH, 657.5 (375.0–1146.3) in mismatch, and 210.0 (118.0–257.5) in no mismatch (*p* < 0.001). V/Q mismatch scores were 0.40 (0.30–0.40) in CTEPH, 0.30 (0.21–0.35) in mismatch, and not available in the no mismatch group (*p* = 0.004).

### 3.2 Hemodynamic and imaging characteristics

The median RV/LV ratio was 0.81 (0.73–0.97) in CTEPH, 0.64 (0.56–0.70) in mismatch, and 0.63 (0.55–0.69) in no mismatch (*p* < 0.001). The median main pulmonary artery (MPA) diameter was 30.5 mm (26.8–34.5) in CTEPH, 25.0 mm (24.0–27.8) in mismatch, and 23.0 mm (21.0–25.5) in no mismatch (p < 0.001). Right atrial pressure (RAP) was 6.00 mmHg (4.00–9.00) in CTEPH, 5.00 (2.50–7.00) in mismatch, and 9.00 (7.00–13.00) in no mismatch (*p* < 0.001). Pulmonary artery wedge pressure (PAWP) did not differ significantly: 9.00 mmHg (6.00–11.00), 8.50 (7.00–9.75), and 10.00 (8.50–10.00), respectively (*p* = 0.193).

Systolic pulmonary artery pressure (sPAP) was 63.0 mmHg (54.5–79.3) in CTEPH, 25.6 (21.5–28.4) in mismatch, and 28.0 (28.0–30.5) in no mismatch (*p* < 0.001). Mean PAP (mPAP) was 38.0 mmHg (29.8–41.3) in CTEPH, 15.6 (14.3–16.9) in mismatch, and 18.0 (17.3–18.8) in no mismatch (*p* < 0.001). Pulmonary vascular resistance (PVR) was 5.50 WU (4.35–7.53) in CTEPH, 1.38 (1.05–1.51) in mismatch, and 1.82 (1.42–1.92) in no mismatch (*p* < 0.001). Cardiac output (CO) was 5.15 L/min (4.56–5.46), 5.67 (4.85–6.55), and 5.20 (4.70–5.50) respectively (*p* = 0.087) (Table 2).

**TABLE 2:** Exploration characteristics of the study population.

Characteristic	CTEPH	No CTEPH with mismatch	No CTEPH nor mismatch	*p* Value
RV/LV ratio (median, IQR)	0.81 (0.73–0.97)	0.64 (0.56–0.70)	0.63 (0.55–0.69)	0.93
MPA diameter, mm (median, IQR)	30.50 (26.75–34.50)	25.00 (24.00–27.75)	23.00 (21.00–25.50)	0.74
RAP, mmHg (median, IQR)	6.00 (4.00–9.00)	5.00 (2.50–7.00)	9.00 (7.00–13.00)	0.12
PAWP, mmHg (median, IQR)	9.00 (6.00–11.00)	8.50 (7.00–9.75)	10.00 (8.50–10.00)	0.193
sPAP, mmHg (median, IQR)	63.00 (54.50–79.25)	25.55 (21.45–28.35)	28.00 (28.00–30.50)	0.22
dPAP, mmHg (median, IQR)	17.00 (15.75–25.25)	10.80 (7.55–13.23)	12.00 (12.00–14.00)	0.34
mPAP, mmHg (median, IQR)	38.00 (29.75–41.25)	15.57 (14.25–16.91)	18.00 (17.33–18.84)	0.41
CO, L/min (median, IQR)	5.15 (4.56–5.46)	5.67 (4.85–6.55)	5.20 (4.70–5.50)	0.087
PVR, Wood units (median, IQR)	5.50 (4.35–7.53)	1.38 (1.05–1.51)	1.82 (1.42–1.92)	0.10

Abbreviations: CO, cardiac output; CTEPH, chronic thromboembolic pulmonary hypertension; MPA, main pulmonary artery; PAWP, pulmonary artery wedge pressure; PVR, pulmonary vascular resistance; RAP, right atrial pressure; RV/LV, right‐to‐left ventricular diameter ratio; sPAP/dPAP/mPAP, systolic/diastolic/mean pulmonary artery pressure.

### 3.3 Time‐averaged wall shear stress (TAWSS) and oscillatory shear index (OSI)

**FIGURE 4:**
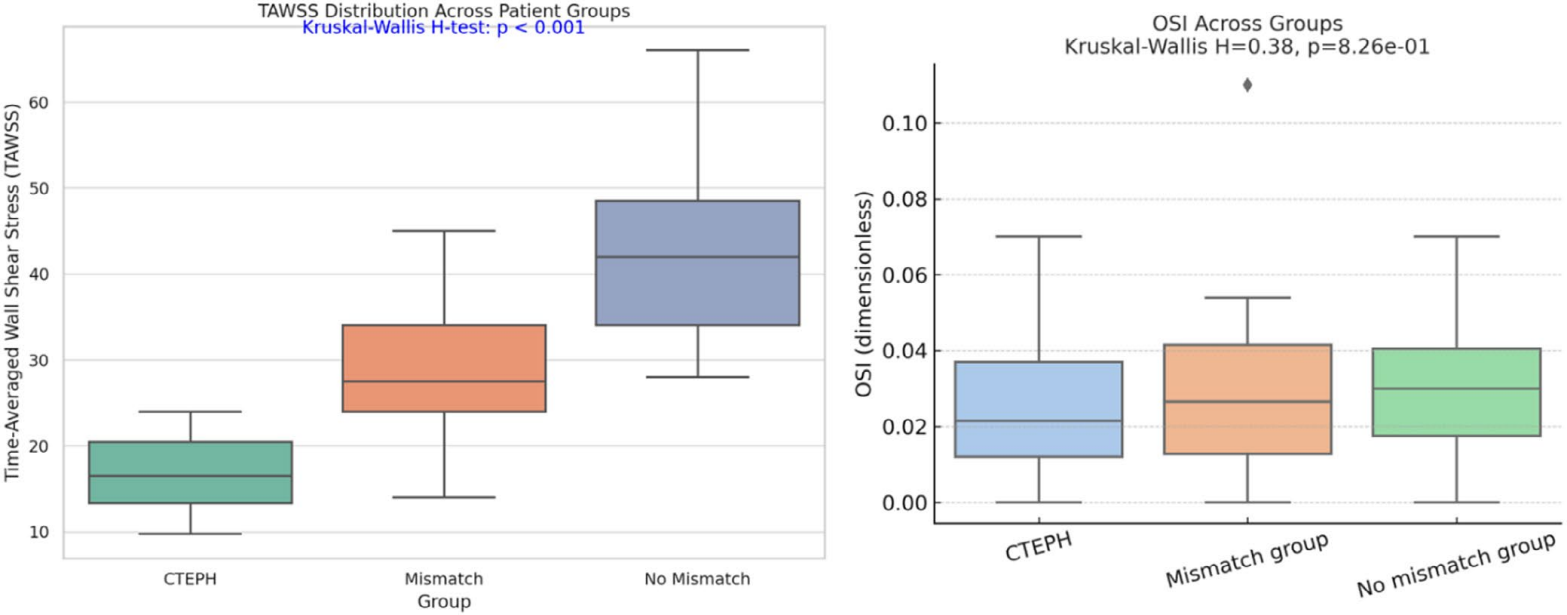
Spatial distribution of time‐averaged wall shear stress and OSI. Time‐averaged wall shear stress (TAWSS) in the left significantly differs across patient groups (*p* < 0.001), with lowest values in CTEPH. Oscillatory shear index (OSI) in the right did not differ significantly (*p* = 0.83). Data shown as boxplots. CTEPH, chronic thromboembolic pulmonary hypertension; OSI, oscillatory shear index; TAWSS, time‐averaged wall shear stress.

Median TAWSS was 16.5 dyn/cm^2^ (IQR 13.31–20.44) in the CTEPH group, 27.5 (24.00–34.00) in the mismatch group, and 42.0 (34.00–48.50) in the no mismatch group. The Kruskal–Wallis H test showed a significant difference across groups (H = 48.27, *p* < 0.001). Pairwise comparisons using Dunn's test with Bonferroni correction yielded adjusted *p* values of 0.00023 for CTEPH versus mismatch, 1.12 × 10^−11^ for CTEPH versus no mismatch, and 0.00120 for mismatch versus no mismatch (Figure 4). Median OSI values did not differ significantly across groups. The Kruskal–Wallis H statistic was 0.38 with a *p* value of 0.826. Dunn's test confirmed no significant differences between any group pairs (all *p* = 1.0).

### 3.4 Association of TAWSS with V/Q mismatch

**FIGURE 5:**
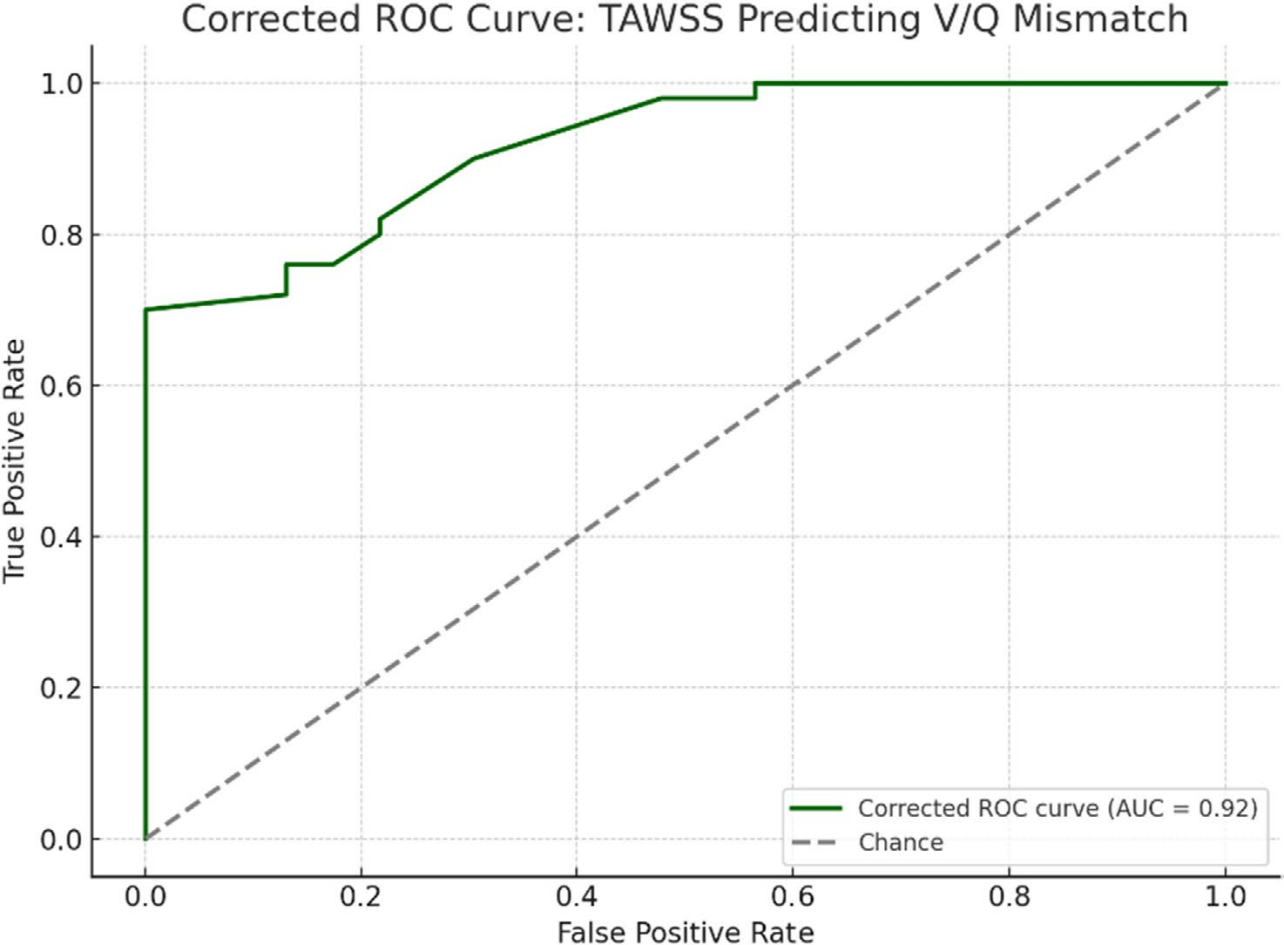
TAWSS correlation and predictive accuracy for perfusion mismatches. Corrected receiver operating characteristic (ROC) curve for TAWSS predicting V/Q mismatch status. The model demonstrates excellent discriminative performance with an area under the curve (AUC) of 0.92. AUC, area under the curve; ROC, receiver operating characteristic; TAWSS, time‐averaged wall shear stress; V/Q, ventilation/perfusion.

Spearman correlation between TAWSS and V/Q mismatch status was *ρ* = −0.673 (*p* < 0.001). ROC analysis of TAWSS for predicting mismatch yielded an AUC of 0.918 (Figure 5). The optimal cutoff for TAWSS was 27.0 dyn/cm^2^, with sensitivity of 70.0%, specificity of 100.0%, positive predictive value of 100.0%, and negative predictive value of 60.5% (Table 3).

**TABLE 3:** Performance of TAWSS to predict mismatch.

Metric	AUC	Optimal TAWSS threshold	Sensitivity	Specificity	Positive predictive value (PPV)	Negative predictive value (NPV)
Value	0.918	27.0	70.0%	100.0%	100.0%	60.5%

Abbreviations: AUC, area under the curve; NPV, negative predictive value; PPV, positive predictive value; TAWSS, time‐averaged wall shear stress.

## 4 DISCUSSION

This study utilized patient‐specific computational fluid dynamics to investigate the potential of hemodynamic parameters, specifically time‐averaged wall shear stress and oscillatory shear index in the proximal pulmonary arteries, to distinguish among patients with CTEPH, those with perfusion mismatches without CTEPH (CTEPD), and individuals without CTEPH or perfusion mismatches. The key finding is a significant difference in median TAWSS across the three groups, with the CTEPH cohort exhibiting the lowest values, followed by the CTED group, and the non‐CTEPH without mismatch group showing the highest TAWSS. In contrast, median OSI values did not differ significantly between the groups. Furthermore, a strong inverse correlation was observed between TAWSS and V/Q mismatch status, and ROC analysis revealed a high area under the curve (AUC) for TAWSS in predicting the presence of perfusion mismatches, suggesting its potential as a noninvasive screening tool.

### 4.1 Hemodynamic alterations and their physiological basis

The observation of reduced TAWSS in the proximal pulmonary arteries of CTEPH patients aligns with findings from numerous previous studies utilizing various approaches, including CFD modeling and phase‐contrast cardiac magnetic resonance imaging (CMR) (Kachabi et al., 2024; Schäfer et al., 2016; Tang et al., 2012). This reduction in shear stress is primarily a consequence of the physical obstruction caused by organized chronic thrombi within the pulmonary arteries, leading to altered blood flow patterns, reduced mean flow velocity, and subsequent luminal remodeling (Gonzalez‐Hermosillo et al., 2024; Simonneau et al., 2017, 2022). Preclinical studies have also supported the link between decreased WSS and vascular remodeling in the pulmonary circulation; for instance, reduced main pulmonary artery (MPA) WSS has been associated with increased collagen and elastin content in a rat model of pulmonary hypertension (Ben Driss et al., 2000). Moreover, disturbed WSS is known to locally attenuate pulmonary endothelial cell nitric oxide release, which could have significant implications for pressure regulation and vascular tone in the pulmonary circulation (Zhou et al., 2014). These findings collectively reinforce the role of altered TAWSS as a biomechanical marker of disease progression in pulmonary vascular pathologies.

### 4.2 Novel insights into chronic thromboembolic disease without pulmonary hypertension (CTEPD)

A significant contribution of our study is the first‐time assessment of TAWSS and OSI in patients with CTEPD (non‐CTEPH with perfusion mismatches). While prior research has extensively documented TAWSS and OSI alterations in established CTEPH (Ben Driss et al., 2000; Schäfer et al., 2016; Tang et al., 2012), the hemodynamic profile of this intermediate patient group remained largely unexplored. The CTEPD group exhibited TAWSS values intermediate between healthy controls and overt CTEPH patients suggesting that these early perfusion defects are associated with a quantifiable, albeit less severe, alteration in proximal pulmonary artery shear stress. This is crucial because CTEPD patients demonstrate persistent anatomical and functional abnormalities that may represent a precursor or milder form of CTEPH. Understanding the biomechanical environment in CTEPD could therefore provide insights into the mechanisms driving disease progression to overt CTEPH, and potentially identify patients at higher risk of future decompensation.

### 4.3 Discrepancy between TAWSS and OSI findings

Interestingly, while TAWSS showed significant differences across our groups, OSI did not differ significantly. This finding contrasts with some prior research that has reported increased OSI in CTEPH patients (Kachabi et al., 2024; Schäfer et al., 2016; Shi et al., 2025; Tang et al., 2012). OSI reflects the degree of oscillation in blood flow direction over a cardiac cycle, and elevated values are generally associated with disturbed flow and endothelial dysfunction. The reasons for the lack of significant difference in OSI in our cohort warrant further investigation. It is possible that the extent and location of chronic thromboembolic disease in our CTEPH group did not consistently result in the specific and widespread proximal flow disturbances that lead to a statistically significant increase in OSI. Given that severe oscillatory flow patterns are often localized to smaller, highly stenotic or occluded vessels, it is plausible that our focus on the proximal pulmonary arteries, while crucial for global flow assessment, might not fully capture the most extreme OSI values that occur more distally. Differences in CFD methodologies, such as the use of patient‐specific 3D models, or variations in patient populations and the specific nature of their thromboembolic burden across different research efforts, could also contribute to these discrepancies. Furthermore, it is conceivable that alterations in TAWSS, reflecting mean flow changes, may precede more altered oscillatory patterns (OSI) in the proximal vasculature during disease development.

### 4.4 TAWSS as a noninvasive screening tool for perfusion mismatches

The strong inverse correlation observed between TAWSS and V/Q mismatch status is a novel and potentially clinically relevant finding. To our knowledge, no previous study has specifically evaluated TAWSS derived from CFD as a predictor for V/Q mismatch presence. V/Q scintigraphy is the recommended initial imaging modality for screening CTEPH due to its high sensitivity in detecting perfusion defects (Humbert et al., 2022; Konstantinides et al., 2020). The significant negative correlation suggests that lower TAWSS values in the proximal pulmonary arteries are associated with a higher likelihood of perfusion abnormalities detected by V/Q scan. The ROC analysis further supports this, with an AUC of 0.918 indicating a high degree of accuracy for TAWSS in distinguishing between patients with and without V/Q mismatches. The optimal cutoff value of 27.0 dyn/cm^2^ for TAWSS yielded a high specificity (100.0%) for predicting mismatch, although the sensitivity was moderate (70.0%). This suggests that while a TAWSS value above this threshold may effectively rule out the presence of perfusion mismatches (high negative predictive value), values below this threshold would necessitate further investigation, potentially indicating the need for V/Q scintigraphy. Physiologically, reduced TAWSS in the proximal arteries likely reflects the altered flow distribution imposed by downstream obstructions or remodeling, even before these changes lead to overt pulmonary hypertension.

### 4.5 Clinical implications

These findings have potentially important clinical implications for the early detection of CTEPH. While V/Q scintigraphy is invaluable, its limitations in terms of cost, availability, and the need for specialized equipment and personnel can hinder its widespread use, particularly in resource‐constrained settings. Our study suggests that CFD analysis of routinely acquired CTPA images, coupled with hemodynamic data from RHC and TTE, could provide a noninvasive means to estimate TAWSS in the proximal pulmonary arteries. A TAWSS value below a certain threshold could then serve as an indicator to prioritize patients for V/Q scintigraphy or referral to specialized CTEPH centres for further evaluation. This approach could potentially improve the efficiency of CTEPH screening and reduce diagnostic delays, which are known to impact patient outcomes (Humbert et al., 2022; Konstantinides et al., 2020).

### 4.6 Limitations

Despite its valuable insights, this study has several limitations. The retrospective nature of the study may introduce selection bias. The relatively small sample size, particularly in the CTEPH group (*n* = 30), may limit the generalizability of our findings. CFD boundary conditions were tuned using RHC data but derived from phase‐contrast CMR of a limited cohort, which may not fully represent individual patient hemodynamics. A further limitation is the use of a rigid wall assumption in CFD modeling, which neglects vascular compliance and may underestimate the dynamic interactions between blood flow and vessel wall motion. Additionally, 3D CFD simulations are computationally intensive and time‐consuming, requiring substantial manual effort for tasks like segmentation, mesh generation, and quality control. This computational burden currently limits the scalability of high‐fidelity simulations for routine use in large clinical cohorts.

### 4.7 Future directions and the role of artificial intelligence

Future research should focus on validating these findings in larger, prospective cohorts of patients with suspected CTEPH. Investigating the role of OSI in different subtypes or stages of CTEPH, potentially incorporating analyses in more distal or specific vessel segments, might also yield valuable insights. Furthermore, exploring the potential for combining CFD‐derived TAWSS with other noninvasive markers, such as echocardiographic parameters or biomarkers like NT‐ProBNP, could enhance the accuracy of CTEPH screening algorithms. Utilizing patient‐specific flow rate curves obtained through phase‐contrast CMR, when available, could also improve the fidelity of the CFD simulations. Advancements in CFD modeling techniques, including the incorporation of fluid–structure interaction and more sophisticated representations of the pulmonary vascular bed, may further refine the clinical utility of hemodynamic parameters in the diagnosis and management of CTEPH.

The complexities associated with patient‐specific model generation and CFD simulation highlight the immense potential benefits of integrating artificial intelligence (AI). AI‐driven automation in tasks such as image segmentation, mesh generation, and even boundary condition tuning could drastically reduce the time and computational expertise currently required. The development of deep learning algorithms capable of rapidly and accurately delineating vascular structures from raw imaging data, or even predicting optimal meshing parameters, would significantly accelerate research in this field. Such advancements would facilitate larger‐scale studies, ultimately enabling the translation of patient‐specific CFD into routine clinical assessment for early CTEPH detection and personalized management, making these powerful tools more widely accessible.

## 5 CONCLUSION

In conclusion, this study showed that CFD‐derived TAWSS in the proximal pulmonary arteries was significantly reduced in victims of pulmonary embolism with CTEPH and those with V/Q mismatches without CTEPH compared to individuals without these conditions. The strong inverse correlation between TAWSS and V/Q mismatch status, along with the high predictive value of TAWSS for identifying perfusion abnormalities, suggests its potential as a novel noninvasive screening tool for CTEPH. Further research is warranted to validate these findings and explore the integration of CFD‐based hemodynamic assessment into the clinical pathway for the early detection and management of this serious condition.

## AUTHOR CONTRIBUTIONS

Identify which authors participated in the research: conceived and designed research, performed experiments, analyzed data, interpreted results of experiments, prepared figures, drafted manuscript, edited and revised manuscript, approved final version of manuscript. The information must be the same as in the online submission site.

## FUNDING INFORMATION

This research did not receive any specific grant from funding agencies in the public, commercial, or not‐for‐profit sectors.

## CONFLICT OF INTEREST STATEMENT

All authors confirm the absence of any relationships with industry relevant to the submitted work.

## ETHICS STATEMENT

This retrospective study was approved by the local Ethics Committee, granting a waiver of informed consent due to the anonymized nature of the data. All procedures adhered to the Declaration of Helsinki and strictly followed French law, including the MR004 reference methodology. Confidentiality was maintained by de‐identifying all patient records.

## Data Availability

The datasets used and analyzed during the current study are available from the corresponding author upon reasonable request. Due to patient confidentiality and ethical restrictions imposed by the Institutional Review Board, the raw data cannot be publicly shared. However, de‐identified data relevant to the findings of this study may be provided upon request, subject to institutional approval and compliance with data protection regulations.

## Shear stress metrics

### Time‐averaged wall shear stress (TAWSS)

The TAWSS is calculated as the time average of the magnitude of the instantaneous wall shear stress (WSS) vector over the cardiac cycle and is given by:

$$\text{TAWSS}=\frac{1}{T}\int_{0}^{T}\left|\tau \left(t\right)\right|\mathrm{dt}$$

where τ(*t*) is the instantaneous wall shear stress vector at time *t*. *T* is the duration of the cardiac cycle.

### Oscillatory shear index (OSI)

OSI quantifies the directional change of the WSS vector during the cardiac cycle and is defined as:

$$\mathrm{OSI}=\frac{1}{2}\left(1-\frac{\left|\int_{0}^{T}\tau \left(t\right)\mathrm{dt}\right|}{\int_{0}^{T}\left|\tau \left(t\right)\right|\mathrm{dt}}\right)$$

where a value of OSI = 0 indicates unidirectional flow. OSI = 0.5 indicates purely oscillatory shear with no net direction.

## Mesh independence study

To ensure numerical robustness and the reliability of computed hemodynamic indices, a mesh independence analysis was performed using a representative patient‐specific pulmonary artery geometry. Three discrete mesh densities—coarse, medium, and fine—were generated within the SimVascular framework using the TetGen meshing algorithm. Global maximum edge lengths of 0.03 cm and 0.02 cm were applied for the coarse and fine meshes, respectively, resulting in approximate element counts of 180,000 (coarse), 350,000 (medium), and over 1.3 million (fine). Simulations were conducted under identical boundary and flow conditions to assess the sensitivity of key flow metrics, including time‐averaged wall shear stress (TAWSS) and pressure drop across the main pulmonary artery.

Mesh convergence was evaluated by quantifying the relative change in these parameters between successive mesh refinements. A difference of less than 5% between the medium and fine meshes was established as the criterion for mesh independence. The coarse mesh exhibited deviations exceeding this threshold and was excluded from further consideration. In contrast, the medium mesh produced hemodynamic outputs closely matching those of the fine mesh, but with substantially reduced computational demands.

Accordingly, the medium‐resolution mesh was selected for all subsequent simulations, providing an optimal balance between numerical accuracy and computational efficiency. This mesh strategy ensured consistent resolution of complex flow features while facilitating high‐throughput simulations across a patient cohort. The use of a validated, mesh‐independent framework enhances the reliability of CFD‐derived biomarkers for assessing pulmonary vascular hemodynamics.

## Simulation lumped parameters tuning process

**Table:**

Parameter	Purpose and physiological role	Initial value and distribution
R_p_ (proximal resistance)	Represents the characteristic impedance of the proximal vasculature immediately downstream of the 3D model.	Initialized using literature‐based impedance ratios (typically 10%–20% of total PVR). Value distributed proportionally to 1/D^2.25^ (Murray law).
C (compliance)	Models the capacitive behavior of the distal pulmonary vasculature, allowing temporary volume storage during systole.	Total compliance estimated from published pulmonary compliance ranges and distributed proportionally to D^2^ across outlets.
R_d_ (distal resistance)	Represents the microvascular resistance of the distal circulation, dominating steady‐state flow opposition.	Calculated as the remaining resistance to match total PVR after assigning R1. Distributed using the same 1/D^2.25^.
Cardiac output (CO)	Represents the total volume of blood pumped by the right ventricle per minute; serves as a key global flow input for CFD.	Obtained from RHC; used to scale the input flow waveform and to calculate total PVR.
RV inflow curve	Time‐varying inflow condition at the MPA inlet; ensures physiologically realistic pulsatile boundary condition.	Estimated using from local cohort waveforms scaled to match cardiac output and heart rate; derived from phase‐contrast CMR
Pressure curves (from RHC)	Provides patient‐specific pressure targets for model calibration; essential for tuning boundary condition parameters.	Measured during RHC (mPAP, sPAP, and dPAP); used to initialize and constrain the Windkessel model fitting process.
